# The Social Context of Cannibalism in Migratory Bands of the Mormon Cricket

**DOI:** 10.1371/journal.pone.0015118

**Published:** 2010-12-14

**Authors:** Sepideh Bazazi, Christos C. Ioannou, Stephen J. Simpson, Gregory A. Sword, Colin J. Torney, Patrick D. Lorch, Iain D. Couzin

**Affiliations:** 1 Department of Zoology, University of Oxford, Oxford, United Kingdom; 2 Department of Ecology and Evolutionary Biology, Princeton University, Princeton, New Jersey, United States of America; 3 School of Biological Sciences, The University of Sydney, Sydney, Australia; 4 Department of Biological Sciences, Kent State University, Kent, Ohio, United States of America; University of Sheffield, United Kingdom

## Abstract

Cannibalism has been shown to be important to the collective motion of mass migratory bands of insects, such as locusts and Mormon crickets. These mobile groups consist of millions of individuals and are highly destructive to vegetation. Individuals move in response to attacks from approaching conspecifics and bite those ahead, resulting in further movement and encounters with others. Despite the importance of cannibalism, the way in which individuals make attack decisions and how the social context affects these cannibalistic interactions is unknown. This can be understood by examining the decisions made by individuals in response to others. We performed a field investigation which shows that adult Mormon crickets were more likely to approach and attack a stationary cricket that was side-on to the flow than either head- or abdomen-on, suggesting that individuals could reduce their risk of an attack by aligning with neighbours. We found strong social effects on cannibalistic behaviour: encounters lasted longer, were more likely to result in an attack, and attacks were more likely to be successful if other individuals were present around a stationary individual. This local aggregation appears to be driven by positive feedback whereby the presence of individuals attracts others, which can lead to further crowding. This work improves our understanding of the local social dynamics driving migratory band formation, maintenance and movement at the population level.

## Introduction

The movement patterns of organisms such as swarming insects, schooling fish or flocking birds can exhibit a level of coordination, cohesiveness and persistence that continues to provide many questions about their mechanism and function. The spatiotemporal patterns that emerge from such groups are often the result of relatively simple interactions occurring between individuals, which ultimately scale to the population level [1], [2]. The decisions made by individuals within a group, for example, regarding where to move, forage, or interact with conspecifics, are governed by their current internal state [3], [4] and sensory information they can receive [5], [6], [7], [8]. Since individuals have a relatively local sensing ability, the decisions they make are typically based on their immediate environment and the behaviour of individuals close enough to be perceived [1], [9], [10].

In western North America the Mormon cricket, *Anabrus simplex*, a flightless katydid, forms huge migratory bands, consisting of up to several million individuals marching together and devouring vegetation [11], [12]. These migratory bands can stretch to over 10 km long and several km wide with individuals capable of marching up to 2 km a day [13], [14], [15], [16]. The mechanism that drives such groups has until recently been poorly understood. Migratory bands appear to function, at least in part, as an anti-predator strategy for individuals within the group. A radiotelemetric mark-recapture experiment revealed crickets that become separated from the band suffered high mortality due to predation [17], hence group cohesion is maintained to minimize predation risk. The individual within the group may also become mobile in order to locate new habitats when resources have become depleted [18].

Sword (2005) showed that stimuli from the immediate presence of conspecifics play an important role in the activation of individual Mormon cricket movement. Crickets have a propensity to cannibalize others, particularly stationary individuals or individuals with reduced mobility, in order to obtain protein and salt [18]. Bazazi *et al.* (2008) revealed that cannibalistic interactions have a strong influence on the marching behaviour of desert locusts. Their laboratory-based study showed that denervation of the locusts' abdomen (reducing the detection of tactile stimuli) and occlusion of visual stimuli from behind significantly decreased marching activity in locust groups and resulted in more cannibalism. Thus cannibalistic interactions or the threat of cannibalism can act to drive marching behaviour as individuals move to reduce their risk of attack from conspecifics [19].

It is likely, therefore, that cannibalism plays a role in the onset and maintenance of coherent swarm motion in Mormon crickets, as suggested by [18]. We hypothesize that individuals can reduce their likelihood of being cannibalized by aligning with others in the band, whereas those that do not align may suffer a higher number of attacks from conspecifics. This risk of being attacked in relation to a cricket's alignment with the group can be quantified by examining the response of band members to stationary individuals that are aligned differently with respect to band direction of travel. Band members must also make decisions about which side of the stationary individual to approach, whether or not to attack once they are close enough, and how long to engage in the encounter. Furthermore, there is natural variation in the flow rate of the band (i.e. local density), which may affect the local social environment and behaviour of crickets.

Ongoing cannibalistic attacks may provide other crickets with information about the location, and quality, of a resource (another cricket), and only simple visual cues such as optical flow and density would be necessary for such assessment [20]. Therefore, it is possible that individuals use cues or inadvertent social information [21] relating to cannibalism in order to reduce uncertainty in the decisions they make. Acquiring social information from the behaviour and performance of others with similar requirements can often be more beneficial than utilizing personal information alone [21], [22], [23].

While there have been many studies on social information use in vertebrates [21], [24], [25], [26], [27], there is increasing evidence that invertebrates, despite often being considered to have less sophisticated information processing capacities, can also use social information [28], [29], [30], [31]. For example, wood crickets modify their predator avoidance behaviour after being exposed to individuals with knowledge of a predator [32]. Flower choice in bumble bees [28] and mate choice in flies [33] are also affected by the preference of others.

The use of social information can lead to not only attraction towards other individuals but can also result in individuals becoming engaged in the same activity as others, known as social facilitation [24], [34], [35]. Therefore the response of individuals to social information (movement towards conspecifics) can act to maintain cohesion in large animal populations [23], [34]. Local aggregations can also lead to strong density fluctuations. This may have important consequences since density has been shown to play a fundamental role in the emergence of collective motion [36].

Here we carried out a field investigation of the behaviour of individual Mormon crickets within migratory bands when faced with a stationary (immobilized) individual. We examine which factors (namely sex, orientation and social influences) are important to cannibalistic interactions in order to shed light on how these simple individual interactions affect the social dynamics driving band formation and maintenance.

### Ethics Statement

All work was carried out in accordance with ethics guidelines and permits.

## Materials and Methods

### Permissions for Fieldwork and Animal Husbandry

All fieldwork was carried out on invertebrates and conducted on public land in the US, therefore no permits were required. No animal husbandry was involved as crickets used in this investigation were obtained from natural populations in the field.

### Study Sites

Experiments were carried out on 15^th^–18^th^ June 2007 within two Mormon cricket bands found at two different sites in Daggett County, Utah, USA (Head of Rye Grass: 40° 47′ 52″N; 109° 8′57″ W; 2220 m; and Lower Rye: 40° 41′ 31″ N, 109° 12′ 13″ W; 2300 m).

There is no pseudoreplication in our data because of the high flow rate of the crickets in these bands, thus effectively our two sites resemble two “laboratories” offering a continuous stream of new subjects. Adult sex ratios were assessed by counting the number of males and females crossing a visualized 1 m transect perpendicular to the flow of the band for 2 min at six separate sites within the bands.

### Experimental Set-up and Protocol

Four immobilized individuals were used in each trial (either all male or all female). To immobilize individuals, each cricket was attached to a thin wooden rectangular block (8×6×0.5 cm) using a cordless butane-powered hot glue gun. A pea-sized amount of glue was placed on the ventral side of the cricket's thorax and abdomen and the individual was glued to the wooden block ventral side down. The crickets were able to move their legs freely as these were not glued down. The four rectangular blocks containing immobilized crickets were placed two body lengths apart (from centre to centre), in a row perpendicular to the migrating band, with each individual orientated in a different direction relative to the direction of flow of the migrating band: right side facing, left side facing, head facing and abdomen (rear) facing. The order of these orientations along the row was randomly determined. Any visible parts of the wooden blocks were covered with soil so that only the immobilized crickets could be observed.

The crickets were then filmed from above using a digital video camcorder (Canon XM2 Digital Video Camcorder) at a rate of 25 frames per second. The first five minutes of each trial provided a habituation period during which band flow could return to normal after setting up the experiment. At the end of the habituation period all immobilized crickets were alive and still possessed all their limbs, which they were able to move freely. The next five minutes were analyzed to examine the interactions between immobilized individuals and approaching crickets from the band. After each trial, crickets and any remaining residue were removed from the wooden blocks, and the blocks were wiped with a dampened cloth. The trials were repeated with different immobilized individuals and their orientations in each position rotated randomly. The time interval between trials was approximately 5–10 min. A total of 24 trials were carried out using 96 immobilized individuals.

### Data Collection

As with predator and prey [37], an interaction begins with an encounter. We define this as when an approaching cricket from the band stopped within one antenna length of an immobilized individual. An encounter may result in the approaching individual moving away and having no further interaction with the immobilized individual. Alternatively, an attack could occur, which itself has two possible outcomes: the attack may be successful (where the approaching cricket bites the stationary individual), or unsuccessful (where the immobilized subject kicks the approacher away). These interactions vary in the intensity of aggressiveness. If during an encounter, more than one interaction was observed, the most aggressive interaction was recorded for that encounter. We consider here a successful attack as being the most aggressive interaction, and movement away from the immobilized subject being the least aggressive interaction. By recording the time each encounter began and ended, the total number of individuals around a focal, immobilized subject could be calculated at each time point. The body part (right side, left side, head or abdomen) of each immobilized individual at which an interaction occurred and the side of the cricket relative to the band flow (directly facing the band, away from the band, left side facing the band, and right side facing the band) were recorded. The flow rate (number of individuals entering the video image) was also recorded, once every ten seconds.

### Statistics

The proportion of encounters and the proportion of successful attacks carried out by male and female approachers were compared using a Wilcoxon signed ranks test. This non-parametric test was carried out since the proportion data were not normally distributed, even after arcsine square-root transformation.

A main-effects Linear Mixed Effects model (LME) was used to analyze effects on the number of encounters, with individual (1 to 96) nested within trial (1 to 24) nested within site as a random factor. Using nested random factors allows us to consider the repeated measures at the individual (multiple encounters with the same individual), trial (multiple individuals within a single trial) and site (multiple trials within a single site) levels. The position of the immobilized individual (a between-individual factor), its sex (between-individual), the body part encountered (within-individual) and the body part relative to the flow (within-individual) were used as explanatory variables. The number of encounters was transformed using a square-root transformation so that the data were normally distributed. The same variables were used to explain variance in the probability that an encounter resulted in an attack, using a main-effects Generalized Linear Mixed Model (GLMM), again with individual as a random variable, nested in trial, nested in site. Finally, the same model structure was also used to analyze the probability that an attack was successful.

A GLMM was used to test whether the probability that an encounter resulting in an attack was affected by the number of individuals present (i.e. the number of ongoing encounters). As multiple encounters occurred per individual, individual identity nested within trials and site were used as the random factor. This method was then repeated to examine whether the number of individuals present affected the probability that an attack was successful or not. A LME, with the same random variable as previously, was used to test the relationship between the number of individuals present and the duration of the encounter. The duration of encounter was log transformed to achieve normality and homoscedasticity.

A cricket's decision to approach an immobilized individual may be affected by the number of individuals present around it (social influence). To detect this, we introduce a ‘Measure of Heterogeneity’ (MOH). This is defined as the observed variance in the number of band members involved in an encounter across each of the four immobilized individuals, normalized by the total number of band members that are engaged in an encounter with any immobilized individual. The result is a metric, invariant to fluctuations in band density, which quantifies the degree of departure from uniformity in the distribution of band members between each of the immobilized individuals. We calculated MOH at every ten seconds in the trials. We also calculated a null case by assuming individuals ‘move’ (i.e. are randomly assigned) to one of the four immobilized individuals independently and with equal probability. For N individuals randomly and independently assigned to M stations, the mean variance between the number of individuals at each station, normalized by N, is found as 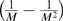. The mean expected null MOH is shown in figures as a grey horizontal line (see Supporting Information S1 for calculations). The observed experimental MOH for all trials was also compared with the random MOH. This was done by dividing the experimental MOH by the random MOH for each experiment every 10 s, and examining whether this ratio was significantly different from 1 using a One-sample t-test. This ratio was square root transformed to achieve normality.

All analyses were carried out in R (version 2.8.0) and Matlab (version 2009b).

## Results

2,056 encounters were observed over the course of the experiment, an average of 4.3 encounters per minute per stationary individual. Of these encounters, 1,258 resulted in attacks (59% of encounters), 734 of which were successful (i.e. the immobilized individual was bitten by an approaching band member; 58% of attacks).

### The behaviours of approachers from the band in response to a stationary individual

**Figure 1:**
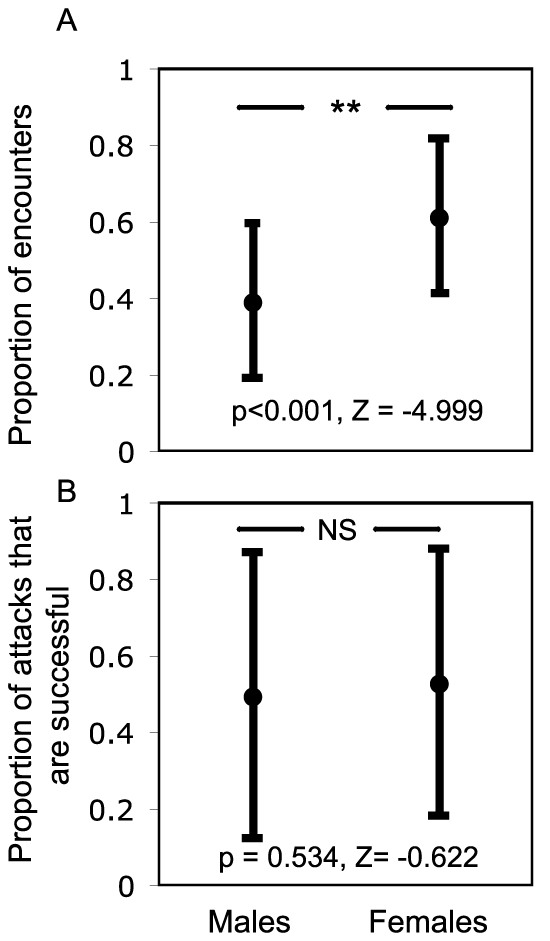
Proportion of encounters and successful attacks by male and female crickets. **A**. The mean proportion of encounters to an immobilized individual carried out by males and females from the band. **B**. The mean proportion of attacks that were successful by males and females. Error bars show +/− one SD. A Wilcoxon signed ranks test was used to compare the means between males and females; the p-values and test statistics are shown.

Overall, the proportion of encounters carried out by females was significantly higher than that of males (Figure 1A, Wilcoxon signed ranks test: p<0.001, Z = −4.999). However, there was no significant difference in the proportion of attacks that were successful between males and females (Figure 1B, Wilcoxon signed ranks test: p = 0.534, Z = −0.622). Sex ratios of adults in the bands were balanced with a mean +/− SEM of 51.8%+/−3.71% and 48.2%+/−3.71% of females in each of the two bands studied.

There was no significant effect of position along the row (one to four) of immobilized individuals on the number of encounters, the proportion of encounters that resulted in attacks, or the proportion of attacks that were successful (LME: F_(3,69)_ = 0.122, p = 0.947; GLMM: F_(3,69)_ = 0.467, p = 0.7064; and GLMM: F_(3,67)_ = 0.866, p = 0.463, respectively). The sex of the immobilized individual also had no influence on the number of encounters to them or the proportion of attacks (LME: F_(1,21)_ = 0.413, p = 0.528; and GLMM: F_(1,21)_ = 0.296, p = 0.5921, respectively). Although not significant, there was a trend for males to experience a slightly higher proportion of successful attacks (GLMM: F_(1,21)_ = 3.00, p = 0.098). The proportion of encounters that resulted in a successful attack was 0.38 for males (of 1066 individuals) and 0.33 for females (of 990 individuals).

**Figure 2:**
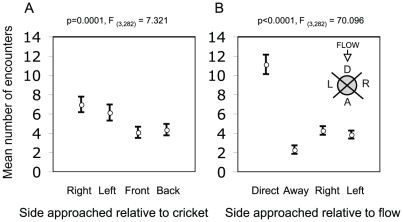
Mean number of encounters. **A**. Mean number of encounters as a function of the side approached to the immobilized individual (right, left, front or back). **B**. Mean number of encounters as a function of the side approached relative to the band flow (direct-directly facing flow, away- furthest away from the flow, right, and left). Inset shows all the positions relative to the flow. Error bars show +/− one SEM.

The right and left sides of the immobilized cricket received the highest number of encounters, compared to the head and abdomen (Figure 2A, LME: F_(3,282)_ = 7.321, p = 0.0001). However this variable did not affect the probability of attack given an encounter, or the success of attacks (GLMM: F_(3,215)_ = 2.50, p = 0.060; and GLMM: F_(3,179)_ = 2.436, p = 0.066, respectively), as all four body parts of the immobilized cricket were equally likely to receive an attack and for that attack to be successful. As expected, the part of the cricket directly facing the band received the highest number of encounters (Figure 2B, LME: F_(3,282)_ = 70.096, p<0.0001). However, encounters were not more likely to result in an attack when facing the flow (GLMM: F_(3,215)_ = 0.814, p = 0.488), and this variable had no significant effect on the probability of successful attacks (GLMM: F_(3,179)_ = 2.436, p = 0.0640).

### The behaviour of approachers from the band in response to the presence of others

**Figure 3:**
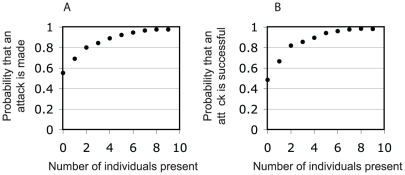
Social influence on attacks. **A**. Probability that an encounter by an approaching band member results in an attack as a function of the number of individuals already present (i.e. within one antennae length) around the immobilized individual. **B**. Probability that an attack by an approaching individual is successful as a function of the number of individuals already present around the immobilized individual.

**Figure 4:**
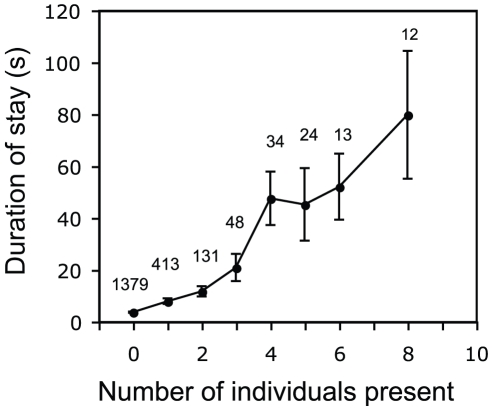
Social influence on the duration of encounter. The duration of encounter (in seconds) of an approaching band member as a function of number of individuals present around the stationary individual when the band member leaves. The numbers of data points used to calculate each mean are shown (N). The mean is not presented for cases where N is less than 10. Error bars show +/− one SEM.

The number of crickets already in contact with the stationary individual had a strong effect on the probability that an approaching cricket from the band attacked the immobilized individual (Figure 3A, GLMM: p<0.0001, z-value = 4.445). Once an attack was made it could either be successful (if the approacher bit the immobilized individual) or unsuccessful (if the approacher was kicked away by the immobilized individual as it approached). The probability of a successful attack by an approacher was also strongly influenced by the number of individuals present around the immobilized individual, increasing as the number of individuals present increased (Figure 3B, GLMM: p = 0.0006, z = 3.464). Furthermore, there was a significant positive relationship between the number of individuals present around an immobilized individual and the duration of stay by the approacher (Figure 4, LME: F_(1,1959)_ = 192.0213, p<0.0001). Hence, encounters lasted longer, were more likely to result in an attack, and were more likely to be successful if other individuals were present around an immobilized individual.

### Heterogeneity and cricket band flow

**Figure 5:**
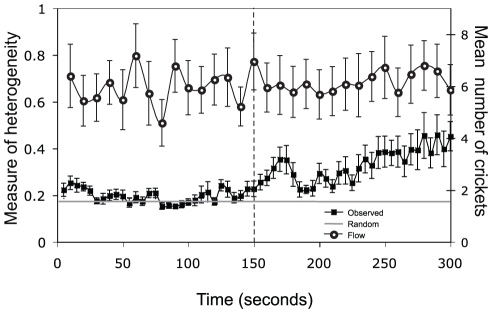
Heterogeneity and flow rate over time (in seconds). MOH for experiments (filled squares) and expected random MOH (grey horizontal line) were calculated for every frame and then averaged over five second intervals for each trial. The mean flow rate from all trials over time was calculated (open circles) every ten seconds. Error bars show +/− one SEM. The vertical dashed line shows the mid-point of the experiment.

The band flow rate and the measure of heterogeneity (MOH) in the number of individuals interacting with immobilized individuals were calculated at every ten seconds of the experiment (Figure 5). The ratio of mean experimental MOH to random MOH was significantly greater than one (the null hypothesis), suggesting that crickets were behaving in a non-random (more clumped) manner (One-sample t-test: p<0.0001, t = 6.1677, Df = 522, mean = 1.407, SEM = 0.4138).

## Discussion

There are three main conclusions arising from our study. First, female Mormon crickets are more likely than males to engage in encounters when approaching stationary individuals within a migratory band, but are no more likely than males to carry out a successful attack. Second, the side of a cricket facing the flow of a migratory band is more likely to be approached by oncoming crickets, with insects whose body axis is perpendicular to the direction of flow being more vulnerable to an encounter than those positioned parallel to band flow direction. Lastly, the duration of individual encounters and the likelihood of a successful attack are strongly affected by the local social environment. These findings provide insights into how individual crickets in the band make decisions, particularly about where to move or forage, and utimately into how the spatiotemporal patterns of migratory bands can emerge [1], [2].

The proportion of females that interacted with a stationary individual was signficantly higher than that of male crickets (Figure 1A) despite there being an even sex ratio within bands and no evidence for a difference in speeds between the sexes based on a concurrently conducted radiotracking study [38]. However, despite female Mormon crickets being larger than males, both females and males were equally successful at attacking (Figure 1B). The propensity of females to more frequently engage in encounters with potential victims of cannibalism may reflect underlying differences in nutritional requirements associated with reproductive investment. Although this seems likely, Mormon crickets are a classic example of sex role reversal in courtship and, in some populations, males must provide a massive spermatophore to females [14]. Further work is required to precisely determine the nutritional targets for males and females in populations of this species.

The body axis orientation of the stationary cricket was also important, with oncoming crickets more likely to approach the left or right side of the stationary individual (Figure 2A). This is perhaps most simply due to the larger observable surface area of the body exposed to contact when oriented as such, and in itself provides a rationale for the collective alignment of band members to minimize the likelihood of an encounter and potentially cannibalistic attack from oncoming crickets. Neither the position nor sex of the stationary individual affected the behaviour of the approaching crickets in our experiments.

The presence of other individuals has a strong influence on the behaviour of crickets in the migratory band: in particular, how likely they are to attack a stationary individual and whether this attack is successful. Individuals have a very high probability of attacking or making a successful attack even in the absence of other individuals (0.55 and 0.49, respectively), but this probability increases, almost doubling once the number of individuals present increases to 9 (0.98 and 0.98, respectively, see Figure 3). Whether or not an approaching band member successfully attacks a stationary individual is also dependent on many other factors, for example the size and age differences between the individuals [39]. Simpson *et al.* (2006) showed that the risk of attack to an individual cricket increases with decreasing mobility and motility. Immobile and immotile insects (glued and dead) were consumed before immobile and motile insects (glued and alive) that were able to kick at their attackers. However, healthy mobile insects were also attacked, and the risk similarly depended on their motility (use of legs to fight off attackers). Thus, the response of attackers to variation in victim motility was in the same direction regardless of whether the victim was immobile or mobile.

Furthermore, as the number of individuals interacting with a stationary individual increases, the duration of the encounter by the approaching cricket also increases. The behaviour of crickets is significantly different to that expected if they were responding randomly with no social influence (Figure 5). The flow of the band did not change over the course of the experiment, suggesting that the increase in the observed MOH (Figure 5) is due to a non-random aggregation of individuals. An increase in the number of individuals interacting with the stationary individual also caused others to stay longer (Figure 4), further enhancing the positive feedback resulting in social enhancement of individual choice. An alternative explanation for the non-random aggregation of individuals could be if they were responding to changes in the behaviour of the immobilized cricket as a result of previous attacks by others, resulting in an indirect social effect on their distribution. Either of these processes appear to amplify density fluctuations in initially homogenous conditions and can be a factor leading to the emergence of local collective patterns in Mormon cricket bands [1], [9].

Our results suggest that social facilitation, an increase in behaviour frequency in response to others engaged in the same behaviour [35], may be occuring since individuals stop to interact with stationary individuals and stay longer with an increasing number of conspecifics already interacting. Social facilitation can often be found in animals, including invertebrates, engaged in feeding activity and typically results in access to resources by some group members that otherwise would not have been able to obtain them [40], [41], [42], [43]. Consistent with this, the number of individuals already interacting with, and potentially injuring, a stationary cricket can provide approaching cannibals with social information about the location of the resource, which they may be less successful at obtaining using personal information alone [21], [22], [34]. Alternatively, cues (such as hemolymph) from the wounded cricket could elicit feeding in approaching individuals. This ‘blood in the water’ effect could explain how the local aggregation around a focal individual forms, as a greater number of crickets are stopping at an increasingly large target and then competing with diminishing returns for access to the corpse. Ultimately both mechanisms result in an equivalent socially mediated positive feedback effect and encourage individuals to move towards other band members.

In summary, social interactions significantly influence the behaviour of individual band members, particularly with regards to aggressive cannibalistic behaviour. Crickets move towards other individuals, in part to obtain essential resources through cannibalism [18], [39] and such social attraction can help maintain group cohesion and can affect the band's collective dynamics. However, while it is known that the presence of conspecifics induces other Mormon crickets to move [44], to date there have been no empirical studies investigating whether individuals actively move towards conspecifics in order to find food, or are simply stopping when they opportunistically encounter a feeding aggregation. Such studies will be important in determining whether Mormon cricket groups form due to active behavioural gregariousness, or alternatively whether they form when individuals are forced to come together due to the patchy distribution of available resources in the habitat.

## Supporting Information

Supporting Information S1 Supporting Information S1 Supporting materials and methods detailing calculations for the random behaviour of crickets to immobilized individuals.
(DOC)
