# Supplementary material for: The Social Context of Cannibalism in Migratory Bands of the Mormon Cricket
Source: PLoS One. 2010 Dec 14;5(12):e15118. doi: 10.1371/journal.pone.0015118 (PMC3001859; doi:10.1371/journal.pone.0015118)
Supplement: Supporting Information S1 — Supporting materials and methods detailing calculations for the random behaviour of crickets to immobilized individuals. (DOC) [file pone.0015118.s001.doc]

**Supplementary Information S1**

**Materials and Methods**

**Calculations for the random behaviour of crickets to immobilized individuals**

Assuming N individuals are randomly and independently distributed between M immobilized individuals, which hereinafter are considered as stations, the mean number of individuals in each station is N/M. If **s** is a vector of the number of individuals in each station so that **s**={s_1_, s_2_…s_M_} and **S** represents the set of all possible permutations, the average between station variance is:

(1)

where the term in square brackets is the variance of a given combination and the second summation averages over all permutations.

Since the order of addition is unimportant, terms may be grouped by station. For station 1 the number of times S_1_=j is equal to (M^N^)Pr(S_1_=j). By rearranging the summation Eqn (1) becomes

(2)

As an individual is equally likely to be placed in any station

(3)

Therefore Eqn 2 defines a binomial distribution, which leads to a variance of

This reduces to , which normalized by N is .
